# Oxytocin-Induced Changes in Intrinsic Network Connectivity in Cocaine Use Disorder: Modulation by Gender, Childhood Trauma, and Years of Use

**DOI:** 10.3389/fpsyt.2019.00502

**Published:** 2019-07-19

**Authors:** Jane E. Joseph, Brandon K. Vaughan, Christopher C. Camp, Nathaniel L. Baker, Brian J. Sherman, Megan Moran-Santa Maria, Aimee McRae-Clark, Kathleen T. Brady

**Affiliations:** ^1^Department of Neuroscience, Medical University of South Carolina, Charleston, SC, United States; ^2^Department of Public Health Sciences, Medical University of South Carolina, Charleston, SC, United States; ^3^Department of Psychiatry and Behavioral Sciences, Medical University of South Carolina, Charleston, SC, United States; ^4^Ralph H. Johnson VA Medical Center, Charleston, SC, United States

**Keywords:** connectome, graph-theory, resting state, gender differences, functional connectivity

## Abstract

Cocaine use disorder (CUD) is a major public health concern with devastating social, economic, and mental health implications. A better understanding of the underlying neurobiology and phenotypic variations in individuals with CUD is necessary for the development of effective and targeted treatments. In this study, 39 women and 54 men with CUD completed a 6-min resting-state functional magnetic resonance imaging scan after intranasal oxytocin (OXY) or placebo administration. Graph-theory network analysis was used to quantify functional connectivity changes caused by OXY in striatum, anterior cingulate cortex (ACC), insula, and amygdala nodes of interest. OXY increased connectivity in the right ACC and left amygdala in males, whereas OXY increased connectivity in the right ACC and right accumbens in females. Machine learning was then used to associate treatment response (placebo minus OXY) in nodes of interest with years of cocaine use and severity of childhood trauma separately for males and females. Childhood trauma and years of cocaine use were associated with OXY-induced changes in ACC connectivity for both men and women, but connectivity changes in the amygdala were associated with years of cocaine use in men and connectivity changes in the right insula were associated with years of cocaine use in women. These findings suggest that salience network nodes (ACC and insula) are potential OXY treatment targets in CUD, with the amygdala as a treatment target for men and the accumbens as a treatment target for women.

## Introduction

Gender differences in addictive and affective disorders are well established (1, 2). Both gonadal and stress hormones can modulate brain function, leading to different levels of susceptibility to neuropsychiatric disorders and treatment response. Biomedical research focused on understanding hormonal modulation and gender differences in brain function may be advanced by including neuroimaging markers of functional brain organization. One such marker is resting-state functional brain connectivity (RSFC), which uses functional magnetic resonance imaging (fMRI) to image the brain while an individual is alert and awake but not engaged in any particular cognitive task; that is, when the brain is at “rest.” This continuous resting-state fMRI (rsfMRI) paradigm can reveal brain regions that are temporally synchronized with other brain regions to characterize brain regions that seem to activate (or deactivate) in unison, revealing additional phenotypes that are not captured with current behavioral assessments or neurobiological markers. Therefore, the addition of rsfMRI as a tool in understanding psychiatric illness and gender-specific susceptibility to different disorders may ultimately lead to better treatments and outcomes.

rsfMRI has been widely used in addictions research, including studies in cocaine use disorder (CUD) (3, 4). Differences in RSFC between CUD and control subjects have been reported in numerous circuits, but there is no clear consensus that any particular circuit or resting-state network can be considered a reliable phenotype for CUD. Nevertheless, RSFC has been associated with important clinical variables, such as measures of cocaine use (5–7), impulsivity, inattention, or cognitive control (5, 6, 8–10) and risk for relapse (10–16). For example, years of cocaine use (which will be the primary cocaine use variable in the present study) have been associated with reduced RSFC in the ventromedial prefrontal, hypothalamic, insula, and anterior cingulate cortex (ACC) regions (7, 14). Although not all studies have shown an association between compromised RSFC and years of use (5), the collective findings point to RSFC as a promising imaging biomarker for relapse risk or other behaviors implicated in the addiction process (17).

However, two important variables that are known to modulate addiction neurocircuitry—gender and trauma exposure—have been less studied in rsfMRI studies of CUD. Sex differences were examined in only one RSFC study (7) and revealed greater connectivity between the medial hypothalamus and a critical node of the default mode network, the precuneus, in female cocaine users compared to males. A recent study has also examined modulation of RSFC by history of childhood trauma in CUD (18). The CUD group reported that some childhood trauma showed greater amygdala RSFC with several striatal regions, the insula, medial temporal regions, and the brain stem. These studies are an important step toward understanding individual differences in RSFC, but more studies are needed to characterize RSFC phenotypes that may lead to the development of individualized treatment approaches.

One potential treatment being explored for substance use disorders (SUD) is the neuropeptide oxytocin (OXY). Childhood trauma (19, 20) and chronic substance use (21) can both lead to neuroadaptations in the OXY system. In addition, some studies have shown that exogenous OXY may reverse drug-induced neuroadaptations [see Ref. (21), for review] or can alter neural response in stress-related circuitry (22–24). However, the effect of exogenous OXY may not be the same in men and women because of gender differences in neuropsychiatric sequelae of childhood trauma and the neurobiology of OXY (25, 26).

Few studies, however, have examined gender differences in RSFC changes caused by acute OXY administration, and no studies have examined these changes in individuals with CUD. Seeley and colleagues (27) reviewed 11 studies that examined changes in RSFC caused by acute intranasal OXY administration in healthy controls and individuals with anxiety disorders (posttraumatic stress disorder, generalized social anxiety disorder) or autism spectrum disorder. Most of these studies focused on connectivity of the amygdala with medial prefrontal or cingulate regions. Although findings are mixed as to whether OXY increases or decreases amygdala connectivity, individual differences like gender and psychopathology modulate this connectivity. Whole-brain analyses of RSFC have indicated that acute administration of OXY also increases connectivity in brain regions other than the amygdala, including the striatum, insula, and cingulated (28, 29). In addition, enhanced connectivity under OXY may depend on gender and trauma history, as well as the specific amygdala (24) or striatal nuclei (30) targeted in a given study.

Prior research has demonstrated that females with SUD associate relapse with interpersonal stress and negative affect (31, 32), whereas males with CUD show a more robust reward circuitry response to cocaine cues than females (33, 34). Potenza et al. (35) reported that corticostriatal-limbic hyperactivity was associated primarily with drug cues in men and stress cues in women. These findings suggest that stress circuitry may play a more important role in intrinsic functional brain organization in women with CUD, whereas reward circuitry may play a more prominent role in men with CUD.

To gain a better understanding of gender differences in neural response to OXY in CUD, the present study used RSFC to examine changes in stress- and addiction-related neurocircuitry in response to an acute dose of intranasal OXY in men and women with CUD. More specifically, the goal of this study was to understand the association between graph-theory-based network properties that reflect OXY treatment response and two individual subject variables of interest for SUD: childhood trauma and years of cocaine use. Predictive modeling was used to establish network profiles of OXY response associated with childhood trauma and years of cocaine use in men and women with CUD. The focus was on network connectivity of regions implicated in both substance use and childhood trauma, that is, the striatum, amygdala, insula, and ACC.

Given prior findings, the predictions of this study were that a) childhood trauma was expected to be more strongly associated with OXY connectivity changes in the amygdala because of its involvement in stress reactivity and trauma history (36, 37) and modulation of amygdala RSFC in posttraumatic stress disorder (PTSD) (24) and recent trauma exposure (38); b) years of cocaine use was expected to be more strongly associated with OXY connectivity changes in the striatum because of neuroadaptations of striatal circuitry in addiction (39); c) the major nodes of the salience network (insula, cingulate) were expected to be associated with both childhood trauma and years of cocaine use because of the role of this network in SUD (17) and psychiatric disorders more broadly (40); d) OXY response in network regions associated with childhood trauma and years of cocaine use was expected to be different in men and women. Prior findings suggest that stress circuitry (e.g., amygdala) will exert a stronger network influence in females and reward circuitry (e.g., striatum) will exert a stronger network influence in males.

## Materials and Methods

### Participants

Participants took part in a large study investigating the effect of OXY on subjective and neuroendocrine responses to stressors. The current crossover analysis included only data from the rsfMRI component of the study. A total of 93 non-treatment-seeking CUD individuals who responded to local media advertisements over a 54-month period completed the fMRI scanning procedures. Written informed consent was obtained before study assessments were administered. All procedures were conducted in accordance with Good Clinical Practice Guidelines and the Declaration of Helsinki and received institutional review board (IRB) approval. General exclusion criteria included 1) pregnancy, nursing, or plan to become pregnant during the course of the study; 2) women who had a complete hysterectomy, were postmenopausal, or receiving hormone replacement or hormonal contraceptive therapy; 3) history of or current significant hematological, endocrine, cardiovascular, pulmonary, renal, gastrointestinal, or neurological diseases; 4) history of or current psychotic, panic, eating, or bipolar affective disorders; 5) current major depressive disorder and PTSD; 6) history of or current medical conditions that might affect hypothalamic pituitary axis (HPA)axis activity; 7) synthetic glucocorticoid or exogenous steroid therapy within 1 month of testing; 8) psychotropic medications (with the exception of selective serotonin reuptake inhibitors), opiates or opiate antagonists, benzodiazepines, antipsychotics, beta-blockers, and other medications that might interfere with HPA axis activity or physiologic measurements; 9) acute illness or fever; 10) *Diagnostic and Statistical Manual of Mental Disorders-IV* (DSM-IV) criteria for substance dependence except alcohol, nicotine, or marijuana within the past 60 days; 11) unwillingness or inability to maintain abstinence from cocaine and other drugs of abuse (except nicotine) for 3 days prior to the cue–reactivity sessions; or 12) MRI contraindications.

### Assessment

Participants meeting prescreening criteria were evaluated for study eligibility with the Mini-International Neuropsychiatric Interview (MINI) (41). The substance use module of the Structured Clinical Interview for DSM-IV (SCID-IV) was used to assess current and lifetime SUD (42). Substance use in the 90 days before the study was assessed using the Time-Line Follow-Back (43). The Childhood Trauma Questionnaire (CTQ) (44) was used to assess the extent to which individuals experienced five domains of childhood abuse and neglect (sexual abuse, physical abuse, emotional abuse, emotional neglect, and physical neglect). Participants answered each of 25 questions using a 5-point Likert scale ranging from 1 (never true) to 5 (very often true). A medical history and physical examination were completed to assess for medical exclusions. Participants meeting inclusion criteria and no exclusion criteria were scheduled to complete the study procedures and instructed to not use cocaine or other drugs of abuse for a minimum of 3 days before the test sessions.

### Study Procedures

Participants completed one 6-min resting-state fMRI session on each of two consecutive days (a cocaine cue reactivity task was also completed on each day, but those results are not reported here). On day 1 of testing, participants arrived at the Medical University of South Carolina’s (MUSC) Addiction Sciences Division research clinic at 10:00 a.m. Upon arrival, urine pregnancy tests were administered. Smokers were provided with a nicotine patch. Self-reports, urine drug screens (Roche Diagnostics, Indianapolis, Indiana), and breathalyzer tests (AlcoSensor III, Intoximeters, Inc., St. Louis, Missouri) were used to assess abstinence. If the pregnancy and drug tests were negative [with the exception of Tetrahydrocannabinol (THC)], study procedures continued. At 11:30 a.m., subjective ratings were obtained. A modified version of the Within Session Rating Scale was used to assess subjective ratings of craving, anxiety, and stress (45). This 1–10 visual analogue scale is anchored with the adjectival modifiers (“not at all,” “mildly,” “moderately,” and “extremely”). The Cocaine Craving Questionnaire (CCQ)-Brief was used to assess cocaine craving. The State-Trait Anxiety Inventory (STAI) was used to assess anxiety symptoms (46). Participants were then provided a standardized lunch.

At 1:20 p.m., participants were administered 40 IU of OXY nasal spray or matching placebo (PBO). This dose was selected based on previous studies using similar doses of OXY (47–49) as well as our own previous work (50, 51). Timing of administration was also based on previous studies showing central activity of OXY 40 min after intranasal administration (50, 52). Intranasal OXY and matching PBO were compounded by the MUSC Investigational Drug Service. To achieve balance in sample size with respect to treatment order across genders, a block randomized design with randomly varying block sizes was used. Half of the participants were randomized to OXY on day 1 and half to PBO.

Subjective measures were repeated at 1:55 p.m. Scanning procedures commenced at 2:00 p.m. The 6-min rsfMRI session instructed participants to fixate a centrally presented crosshair but otherwise had no specific instructions other than to remain awake and alert and minimize head movement.

fMRI data images were acquired on a Siemens Trio 3.0 Tesla scanner with a 12-channel head coil (Siemens Medical, Erlangen, Germany) at MUSC for the majority of subjects (36 females, 53 males). Data from four of the subjects (one male) were collected on a Siemens PRISMA FIT 3.0 Tesla scanner with a 32-channel head coil, also at MUSC. During initial scanner tuning, localizing, and structural scanning, participants were shown relaxation images (i.e., 20 scenic pictures, each displayed for 30 s, and repeated if necessary). A high-resolution T1-weighted MPRAGE anatomical scan (TR = 2.25 s, TE = 4.2 ms, flip angle = 9°, 176 sagittal slices, field of view = 256 mm, 256 × 256 matrix, thickness = 1.0 mm) covering the entire brain and positioned using a sagittal scout image was acquired for coregistration and normalization of functional images. T2*-weighted gradient echo EPI images were acquired with the following parameters (parameters were identical for the TRIO and PRISMA): TR = 2,000 ms, TE = 27 ms, flip angle = 76°, 36 axial slices (field of view = 237 mm × 237 mm, thickness = 3.7 mm voxels, in interleaved order). A gradient field map image was collected to match the spatial parameters of the EPI images.

After completion of the first scan, participants returned the next day and completed identical procedures with the opposite treatment condition. At the end of the second scan day, participants were debriefed and compensated.

### Data Analysis

#### Demographics and Subject Characteristics

Baseline demographic and subject characteristics as well as prescan subjective ratings were compared across genders using independent-samples *t*-tests for continuous variables and chi-square tests across categorical characteristics. Data are reported as means and standard deviations for continuous variables and proportions for categorical variables.

An independent-samples *t*-test (unequal variances assumed because of unbalanced sample sizes) compared PBO minus OXY difference score for TRIO versus PRISMA scanner data in each of the 20 nodes of interest for clustering coefficient (CC) or eigenvector centrality (EC). Significance was determined using the false discovery rate (FDR) controlled at a 5% level (53, 54). Similarly, an independent-samples *t-*test (assuming unequal variances) examined whether PBO minus OXY difference score was different for smokers versus nonsmokers in each of the 20 nodes of interest for CC or EC.

Although several measures were taken to minimize the contributions of head motion to the fMRI time series, there are more stringent approaches to control for the influence of head motion on fMRI time series (55) than used here. To address whether any residual head motion was correlated with graph-theory measures of connectivity, we examined Spearman-rank correlations between head motion and any of the 20 nodes × 2 graph-theory measures (EC and CC) × 2 genders × 2 treatment conditions (OXY or PBO) using FDR correction.

Finally, an exploratory analysis examined whether any of the five subjective rating measures collected before scanning on each visit (craving, anxiety, stress, STAI, CCQ) was correlated with graph theory measures. Spearman rank correlations were conducted for each of the five subjective measures × 20 nodes × 2 graph-theory measures (CC and EC) × 2 genders × 2 treatment conditions (OXY or PBO) using FDR correction.

#### fMRI Preprocessing

FMRIB’s FSL package^1^ was used unless otherwise noted. Images in each participant’s time series on each day were corrected for geometric distortion and head motion. Slice timing correction and spatial filtering (FWHM = 7.5 mm) were applied to each time series, which was then submitted to multiple regression using FSL to remove effects of global signal and head motion. Regressors included global signal [extracted from gray matter, white matter and cerebrospinal fluid (CSF) masks, which were created using FSL’s FAST tissue segmentation tool], and six head motion parameters. The residual image from this regression step was then band-pass filtered (0.009 to 0.08 Hz) using AFNI (56). The spatially normalized image was then parcellated using a 294 region atlas—the 264 regions from Power et al. (57) with 30 additional subcortical regions (amygdala, hippocampus, striatum). Each region of interest (ROI) was represented by a 10-mm-diameter sphere. The BOLD signal time series was extracted in each of the 294 ROIs using FSL’s “feat query” function.

#### Connectome Measures

Before computing the 294 × 294 functional connectivity matrix, corrupt time points were identified with fractional displacement values using the “fsl_motion_outliers” command. For each corrupt time point, the preceding time point and two successive time points were removed from the time series for each subject and visit (57) using the RSFC Net toolbox^2^ implemented using the R software package (58). The mean percent scrubbed time points averaged over both visits was not significantly different between males (M = 0.13, SD = 0.06) and females (M = 0.12, SD = 0.06) according to an independent-samples t-test, t(91) = 0.56, p = 0.58.

The connectivity matrix was a weighted, signed adjacency matrix representing a fully connected undirected graph. Each matrix element reflected the partial correlation between two discreters fMRI time series while controlling for all other time series. We applied a shrinkage factor as to create a well-conditioned covariance matrix (59–61)^3^. The mixing parameter is largely an optimal weight as a function of *N* to combine the observed covariance and a target matrix, such as a diagonal (i.e., no covariance/correlation between regions).

The RSFC Net toolbox was used to compute two graph-theory measures: EC and CC. EC is a spectral, self-referential measure of centrality (62, 63). A node with a high EC is connected to other nodes with a high eigenvector score. EC considers connections to influential nodes to be more important than connections to marginal nodes. Hence, EC reflects the *global influence* of a node on the network.

$$C_{Eig[i]}=\left|\frac{1}{\lambda^{\prime }}\sum_{j=1}^{N}M_{i,j}x_{j}\right|$$

The eigenvector centrality of the *i* ^th^ node, *C* *_Eig_*_[_ *_i_*_]_, is defined as the absolute value of the *i* ^th^ number in the eigenvector belonging to the principal eigenvalue of the matrix *M*, which is denoted λ′.

CC is a local measure of segregation representing the fraction of a node’s neighbors that are also neighbors of each other; these patterns effectively form triangles around the node (64–66). We used the CC formula for weighted and signed connectivity matrices provided by (66):

$$CC_{i}=\frac{\sum_{i,j}w_{s(j,i)}w_{\left(i,q\right)}w_{s(j,q)}}{\sum_{i\neq j}\left|w_{s(j,i)}w_{s(i,q)}\right|}$$

CC reflects the degree of *local influence* in a network. In this formula, the triangle is denoted by the direct connection of the *i* ^th^ and *j* ^th^ nodes and an indirect connection through a *q* ^th^ node; *s*(*i,j,q*). The numerator is the sum of the products of the signed edge weights between the pairs *s*(*i,j*), *s*(*i,q*), and *s*( *j,q*) divided by the sum of the absolute value of the product of the edge weights for pairs *s*( *j,i*) and *s*(*i,q*). The denominator represents the maximum magnitude of the value the numerator can obtain.

EC and CC measures were chosen because they reflect different aspects of network organization. Network measures were always calculated using all 294 nodes. Visualization of nodes used BrainNet Viewer (67).

Twenty nodes were used as ROIs in subsequent analyses (**Table 1**): five insula regions, five ACC regions, six amygdala regions, and four striatal regions. ROIs were selected based on being strongly implicated in addiction (3, 17) and trauma (68–71). Of the eight ACC regions available in the Power atlas, two that fell on the midline were eliminated and five of the remaining six that sampled different aspects of the rostral to dorsal gradient were chosen. Of the seven insula regions available in the Power atlas (only two in the left hemisphere), five were chosen that sampled anterior, mid, and posterior aspects of the insula, primarily in the right hemisphere as there were more of those in the Power atlas. All six amygdala, two accumbens, and two caudate regions were selected. Importantly, the network measures reflected the connectivity of a given node with all other nodes in the whole brain network, not just the connectivity among the 20 nodes of interest.

**Table 1:** Twenty regions of interest used as predictors.

Region name	MNI coordinate		
	x	y	z
Right dorsal ACC	10	−2	45
Right posterior insula	36	−9	14
Right mid insula	37	1	−4
Left ACC	−5	18	34
Left rostral ACC	−11	45	8
Right rostral ACC	12	36	20
Left anterior insula	−35	20	0
Right anterior insula	36	22	3
Right anterior ventral insula	34	16	−8
Right ACC	10	22	27
Left dorsal amygdala	−22	−4	−12
Right dorsal amygdala	22	−4	−12
Left medial amygdala	−14	−4	−20
Right medial amygdala	14	−4	−20
Left ventrolateral amygdala	−28	−4	−22
Right ventrolateral amygdala	28	−4	−22
Left caudate	−13	7	10
Right caudate	14	8	11
Left nucleus accumbens	−10	12	−7
Right nucleus accumbens	10	10	−8

ACC, anterior cingulate cortex; MNI, Montreal Neurological Institute.

#### Generalized Linear Model Analysis (Analysis 1)

The purpose of this analysis was to isolate regions that showed effects of OXY treatment and establish that changes in connectivity caused by OXY were modified by gender, childhood trauma (CTQ), and years of cocaine use (YRSUSE).

Generalized linear mixed effects models were developed to assess Analysis 1 (IBM SPSS tatistics; Version 24.0; IBM Corp., Armonk, NY). Models were developed to specifically assess the effects of treatment (OXY, PBO) and node (20 ROIs described above) as repeated effects, with gender, head motion, CTQ, and YRSUSE as additional variables. All models further adjust for study-specific design variables, specifically study visit and treatment order. To assess the hypothesis that gender, CTQ, and YRSUSE may modify the relationship between OXY and node response, model interactions were included in subsequent analysis. Both main effects and interactions were considered significant if *p* ≤ 0.05. Separate generalized linear models were conducted with CC and EC as outcome variables. This step was conducted before model selection (Analysis 2) to investigate and establish important interactions among variables of interest. Analysis 2 will then examine such interactions in more depth using model selection.

#### Automatic Linear Modeling (Analysis 2)

The purpose of this analysis was to conduct model selection to select the best set of brain regions and network properties associated with differing levels of childhood trauma and years of cocaine use. Eight different models were examined based on the combination of two different outcome variables (CTQ, YRSUSE), two genders (male, female), and two different network measures (CC, EC). For each of the eight models, model selection was conducted over 10 replications.

Model selection used Automatic Linear Modeling (ALM; IBM SPSS Statistics). ALM is a linear modeling approach in which a set of variables (i.e., network properties in each of the 20 ROIs) predicts an outcome (i.e., CTQ or YRSUSE). The treatment effect was expressed as a difference score in either CC or EC in the PBO condition minus the OXY condition in each of the 20 ROIs. A positive difference score reflected a *reduction* in connectivity because of treatment with OXY, whereas a negative difference score reflected *increased* connectivity because of OXY. ALM automatically trims outliers and transforms variables, if needed. ALM divides the full sample of subjects into a training set (70% of the data) and a test set (30% of the data; called the overfit prevention set in IBM SPSS Statistics). The modeling process used 10 replicated data sets, and training and test sets are randomly selected from each. Replicates were a random sample with replacement.

In ALM, if the number of predictor variables is 20 or fewer, a large subset of possible models is examined using “best subsets” (72). This approach determines the best subset of predictor variables using the average squared error (ASE) of the test set. The model with the lowest ASE is chosen by ALM as the best model. ALM yields a measure of model accuracy, which is 100 times the adjusted *R* ^2^ of the final model, Akaike’s Information Criterion (AIC), as well as the importance and weight (coefficient) of each predictor.

Predictor importance is a relative measure of how important each variable was in the prediction. IBM SPSS Statistics uses the leave-one-out method to compute importance based on the residual sum of squares by removing one predictor at a time from the final full model. The importance values all sum to 1.

To determine whether EC or CC yielded a better model for predicting CTQ or YRSUSE for males and females separately, the average accuracy across the 10 replications were compared qualitatively, and the number of significant models (*p* ≤ 0.05) across the 10 replications was considered. The network measure that yielded the highest average accuracy and more significant replications for a given gender and outcome variable combination was considered the better model. To determine the final set of predictors, the cumulative importance of predictors across the 10 replications was calculated. Predictors with cumulative importance >1 were considered for interpretation. Finally, to address potential collinearity among the predictors in the final models, the predictors with cumulative importance >1 were entered into a simultaneous linear regression, and variance inflation factors (VIFs) were determined for each model covariate; if a VIF exists greater than 4.0 (73), multicollinearity will be mitigated by choosing the collinear variable that produces the greatest model fit when included.

## Results

### Demographics and Subject Characteristics

Males were older than females and reported more years of cocaine use (**Table 2**). However, males and females were not different on any of the other demographic, cocaine use characteristics, or subjective measures. There were no significant differences between TRIO and PRISMA scanner data in any of the 20 nodes of interest for either CC or EC. There were also no significant differences between smokers and nonsmokers in any of the 20 nodes of interest for either CC or EC. Therefore, scanner type and smoking status were not included as variables in subsequent analyses.

**Table 2:** Demographics and subject characteristics.

Characteristic	Sex		*p* value
	Female	Male	
	(*n* = 39)	(*n* = 54)	
Demographics			
Age in years (SD)	40.0 (8.5)	44.5 (9.8)	0.024
Cigarette Smoker % (*n*)	84.6 (33)	75.9 (41)	0.305^a^
Cigarettes per day (SD)	11.5 (6.9)	10.8 (6.9)	0.715
Caucasian % (*n*)	30.1 (12)	22.2 (12)	0.352^a^
Cocaine use characteristics			
Age at first use (SD)	22.1 (5.8)	21.1 (6.3)	0.427
Total years use (SD)	14.1 (7.7)	18.3 (8.2)	0.014
Age at dependence onset^b^ (SD)	29.2 (8.1)	29.5 (8.7)	0.849
Using days per month (SD)	17.5 (8.1)	17.0 (7.4)	0.753
Baseline trauma			
CTQ total score^c^ (SD)	51.2 (21.4)	43.8 (14.3)	0.079
Prescan subjective ratings—Visit 1			
Craving (SD)	2.3 (2.7)	2.7 (2.5)	0.563
Anxiety (SD)	2.4 (2.4)	2.3 (2.2)	0.857
Stress (SD)	1.5 (2.3)	2.2 (2.4)	0.167
STAI (SD)	32.2 (9.7)	35.2 (12.1)	0.210
CCQ (SD)	5.5 (1.3)	5.5 (1.1)	0.981
Prescan subjective ratings—Visit 2			
Craving (SD)	2.2 (2.5)	2.5 (2.5)	0.534
Anxiety (SD)	2.0 (2.4)	2.1 (2.5)	0.726
Stress (SD)	1.5 (2.4)	1.7 (2.2)	0.747
STAI (SD)	32.4 (12.0)	34.4 (12.0)	0.430
CCQ (SD)	5.7 (1.3)	5.6 (1.2)	0.677

SD, standard deviation; STAI, State-Trait Anxiety Inventory; CCQ, Cocaine Craving Questionnaire.

a p value calculated using chi-square test.

b Based on responses from 37 females and 53 males.

c Based on responses from 36 females and 49 males.

Head motion was not correlated with CC or EC in any of the 20 nodes or treatment conditions. Although none met the threshold for significance, head motion was included in the two primary analyses below as a precaution given that only six head motion parameters were used as nuisance variables in preprocessing.

Finally, the exploratory correlation analysis between subjective ratings and graph theory measures yielded one significant correlation: males in the PBO condition who reported higher stress before scanning also showed higher EC in the left dorsal amygdala, *rho* = 0.53, *p* = 0.000046.

*Analysis 1:* Establish whether graph-theory measures reflecting treatment response are associated with childhood trauma and years of cocaine use and whether gender moderates these associations.

**Figure 1:**
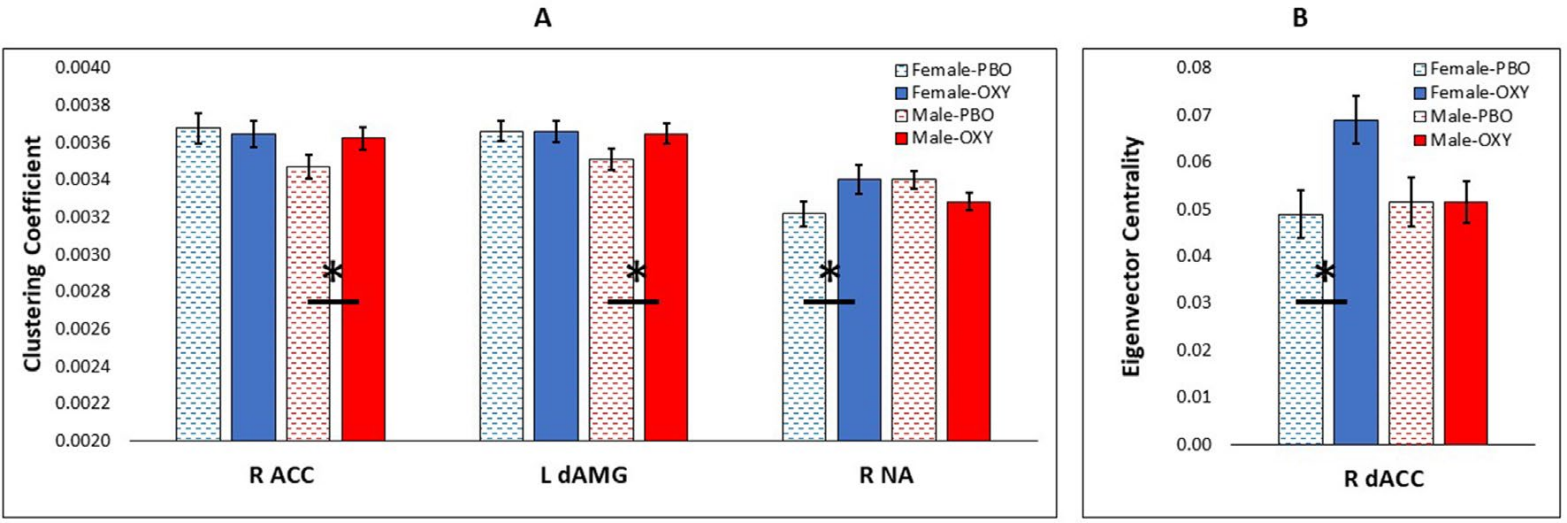
Significant effect of oxytocin (OXY) treatment (solid bars) versus placebo (PBO) (shaded bars) in CUD females (blue) and males (red). **(A)** Effect of OXY on clustering coefficient in three nodes of interest: right anterior cingulate cortex (R ACC), left dorsal amygdala (L dAMG), and right nucleus accumbens (R NA). **(B)** Effect of OXY on eigenvector centrality in one node: right dorsal ACC (R dACC). Error bars are standard error of the mean. Horizontal bars with asterisk indicate a significant difference of OXY versus PBO at *p* < 0.05.

The generalized linear model with CC as the outcome variable and node, treatment, gender, head motion, CTQ, and YRSUSE as predictors yielded several significant effects and interactions (**Supplement 1**). CC varied by node (*p* = 0.0001), CTQ (*p* = 0.009), and head motion (*p* = 0.0001). The node effect was further modified by treatment (Node × Treatment interaction, *p* < 0.0001), and significant three-way interactions indicated that the treatment effect in different nodes was further modified by gender (Node × Treatment × Gender, *p* = 0.0001), CTQ (Node × Treatment × CTQ, *p* = 0.0001), and YRSUSE (Node × Treatment × YRSUSE, *p* = 0.0001). **Figure 1A** illustrates the Node × Treatment × Gender interaction for CC. OXY increased CC for males in the right ACC and left dorsal amygdala, whereas OXY increased CC for females in the right accumbens.

The generalized linear model with EC as the outcome variable and node, treatment, gender, head motion, CTQ, and YRSUSE as predictors yielded a main effect of node (*p* = 0.0001) and higher-order interactions with node (**Supplement 1**). The node effect was further modified by treatment and gender (Node × Treatment × Gender, *p* = 0.0001), treatment and CTQ (Node × Treatment × CTQ, *p* = 0.0001), and treatment and YRSUSE (Node × Treatment × YRSUSE, *p* = 0.0001). **Figure 1B** illustrates the Node × Treatment × Gender interaction for EC. OXY increased EC for females in the right dorsal ACC.

These analyses modeled the spatial correlation among the 20 nodes and isolated treatment effects in some of the nodes. For both EC and CC, these treatment effects were modified by gender, CTQ, and YRSUSE. The goal of the next analysis was to use model selection and machine learning to establish the network profiles associated with OXY-related changes in connectivity measures and CTQ or YRSUSE. Because gender modified these effects in Analysis 1, these analyses are conducted separately in males and females.

*Analysis 2:* Conduct model selection to select the best set of brain regions and network properties associated with childhood trauma and years of cocaine use.

**Table 3** summarizes the performance of the 10 replications for each of the 8 models.

**Table 3:** Model accuracy (adjusted *R* ^2^, top row) and *p* value (bottom row) for each replication for each model of interest.

			Replication										
Outcome Variable	Gender	Graph-theory Measure	1	2	3	4	5	6	7	8	9	10	Mean
CTQ	Male	CC	12%	7%	14%	1%	9%	7%	2%	19%	11%	13%	9%*
			0.11	0.26	0.09	0.40	0.10	0.22	0.36	0.03	0.09	0.07	
	Male	EC	0%	0%	1%	0%	0%	0%	3%	2%	3%	0%	1%
			0.68	0.60	0.42	0.58	0.42	0.79	0.29	0.38	0.31	0.60	
	Female	CC	15%	13%	15%	15%	9%	0%	11%	6%	6%	3%	9%
			0.06	0.17	0.11	0.11	0.17	0.52	0.20	0.23	0.34	0.36	
	Female	EC	28%	29%	39%	37%	23%	35%	31%	24%	21%	7%	27%*
			0.04	0.02	0.01	0.01	0.03	0.01	0.01	0.03	0.03	0.29	
YRSUSE	Male	CC	17%	5%	6%	2%	7%	15%	2%	0%	21%	4%	8%
			0.05	0.18	0.26	0.36	0.17	0.11	0.34	0.64	0.03	0.29	
	Male	EC	18%	23%	19%	10%	15%	17%	27%	3%	1%	22%	16%*
			0.03	0.01	0.01	0.07	0.06	0.03	0.01	0.34	0.30	0.02	
	Female	CC	6%	24%	11%	30%	24%	25%	14%	28%	21%	21%	20%*
			0.23	0.01	0.16	0.01	0.02	0.02	0.10	0.01	0.03	0.05	
	Female	EC	13%	21%	16%	37%	40%	34%	4%	6%	14%	11%	19%
			0.16	0.06	0.06	0.01	0.01	0.02	0.34	0.27	0.09	0.18	

*Indicates best model based on average accuracy and number of significant replications when comparing EC and CC.

CTQ, Childhood Trauma Questionnaire total score; YRSUSE, years of cocaine se; CC, clustering coefficient; EC, eigenvector centrality.

### Network Profile for CTQ in Males

In males, neither the CC nor the EC model was associated with CTQ reliably across replications. Only one replication was significant for CC, and no replications were significant for EC. These results indicate that OXY-related changes in graph-theory measures in the 20 nodes of interest are not associated with individual variations in CTQ scores in males.

### Network Profile for CTQ in Females

In females, the best model for CTQ was based on EC. Across 10 replications, this model had an average adjusted *R* *^2^* of 0.27. Nine of the 10 replications yielded significant models. The model using CC as the graph-theory metric for CTQ had an average adjusted *R* *^2^* of 0.09, and none of the replications was significant.

**Figure 2:**
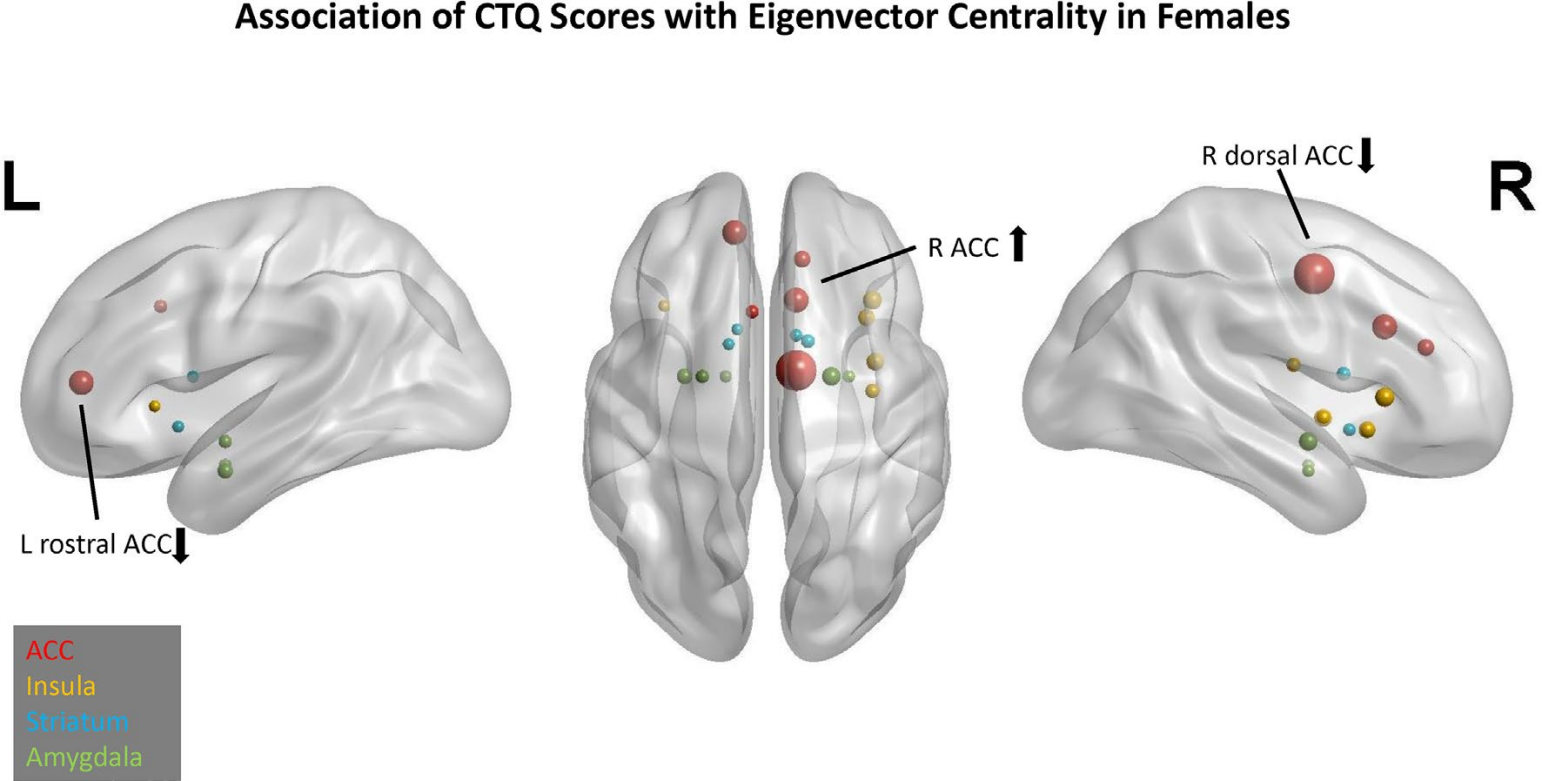
Association of Childhood Trauma Questionnaire (CTQ) scores with eigenvector centrality in females. Network nodes of interest are shown on a template brain in Montreal Neurological Institute (MNI) space, with left lateral (left panel), right lateral (right panel), and axial views (center). Anterior cingulate (ACC) nodes appear in red, insula nodes in yellow, striatum nodes in blue, and amygdala nodes in green. The size of each node reflects its cumulative importance across 10 replications of predictive modeling. Nodes with cumulative importance >1 are labeled anatomically. The arrow next to each label indicates the sign of the regression coefficient for that node. Nodes that failed to appear in any of the 10 replications do not appear in this figure.

In the EC model, three predictors had cumulative importance >1 (**Figure 2**). The scatter plots (**Supplement 2**) illustrate that for the right ACC, a higher CTQ was associated with a greater global influence on PBO than OXY, but for the right dorsal ACC and left rostral ACC, a higher CTQ was associated with a greater global influence on OXY than PBO.

### Network Profile for YRSUSE in Males

In males, the best model for YRSUSE was based on EC. Across 10 replications, this model had an average adjusted *R* *^2^* of 0.16. Six of the 10 replications yielded significant models. In contrast, the model using CC as the graph-theory metric for YRSUSE had an average adjusted *R* *^2^* of 0.09 and only two replications were significant.

**Figure 3:**
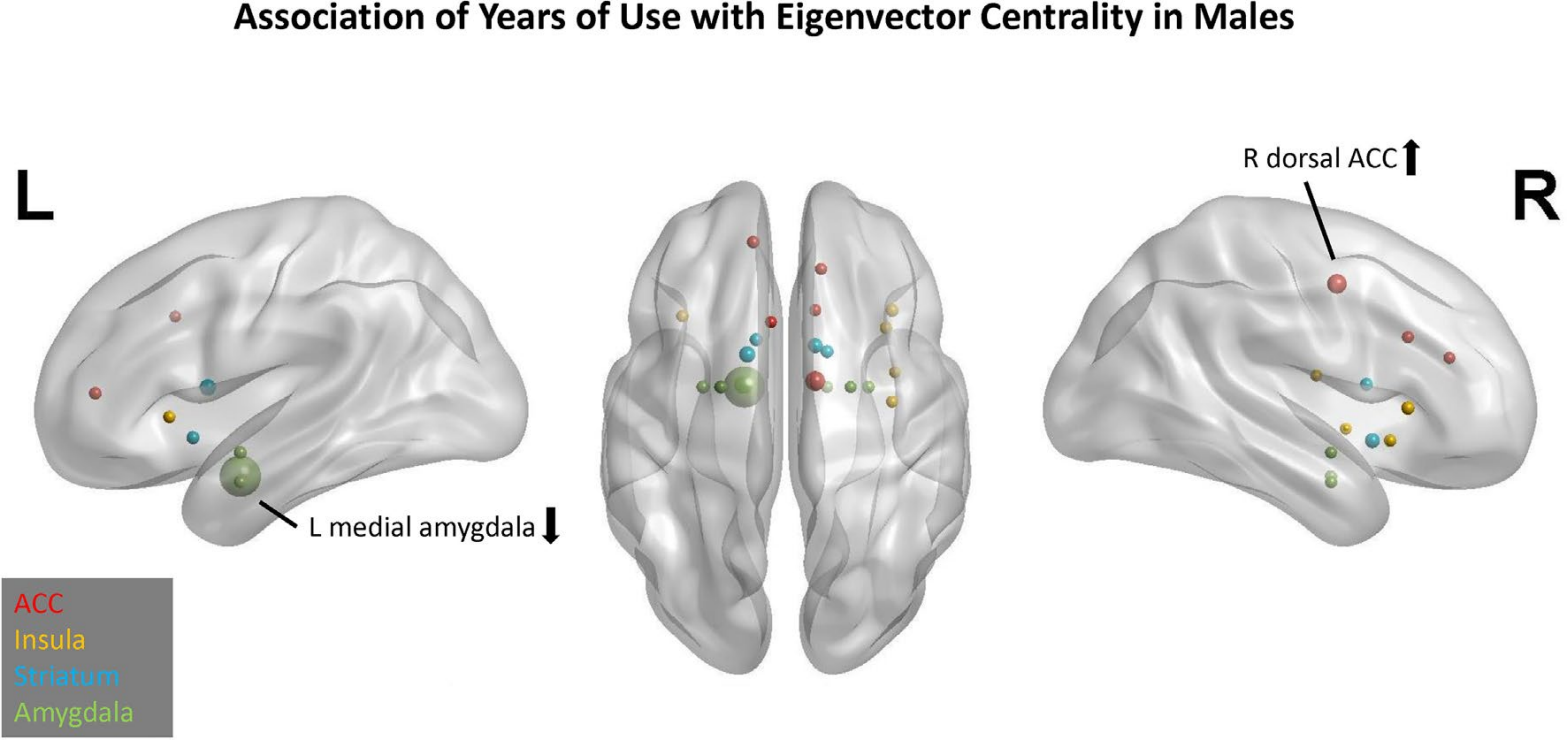
Association of years of cocaine use with eigenvector centrality in males. Network nodes of interest are shown on a template brain in MNI space, with left lateral (left panel), right lateral (right panel), and axial views (center). ACC nodes appear in red, insula nodes in yellow, striatum nodes in blue, and amygdala nodes in green. The size of each node reflects its cumulative importance across 10 replications of predictive modeling. Nodes with cumulative importance >1 are labeled anatomically. The arrow next to each label indicates the sign of the regression coefficient for that node. Nodes that failed to appear in any of the 10 replications do not appear in this figure.

In the EC model, three predictors had cumulative importance >1 (**Figure 3**). The scatter plots (**Supplement 2**) illustrate that for the right dorsal ACC, higher CTQ was associated with greater global influence on PBO than OXY, but for the left medial amygdala, higher CTQ was associated with a greater global influence on OXY than PBO. Greater head motion was associated with fewer years of cocaine use.

### Network Profile for YRSUSE in Females

In females, the best model for YRSUSE was based on CC. Across 10 replications, this model had an average adjusted *R* *^2^* of 0.20. Seven of the 10 replications yielded significant models. Although the model using EC as the graph-theory metric for CTQ had an average adjusted *R* *^2^* of 0.19, only three of the replications were significant. Although the two models had comparable accuracy, the models using CC as a predictor had more replications that were significant, so it was considered a better model than the EC model.

**Figure 4:**
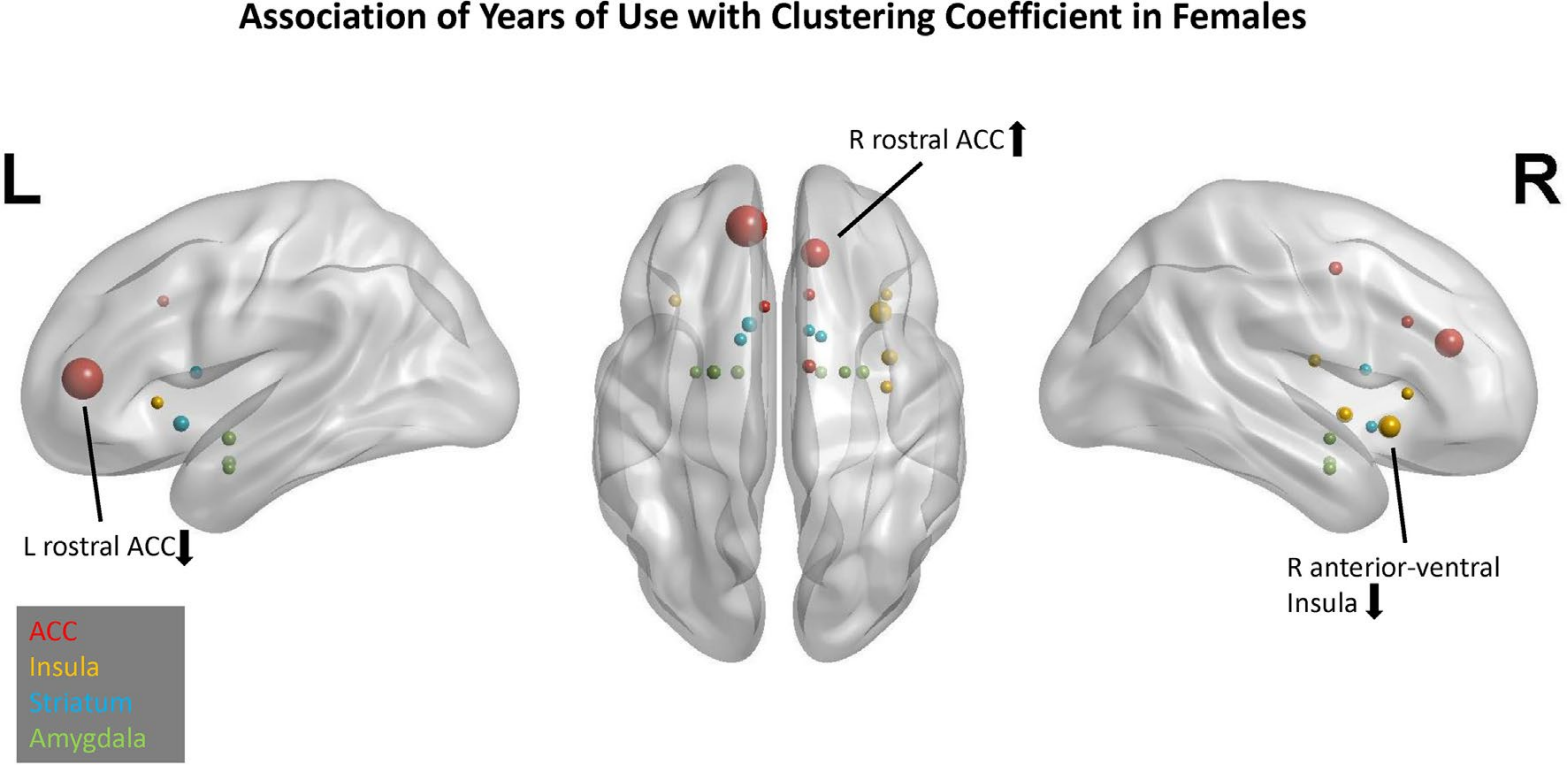
Association of years of cocaine use with clustering coefficient in females. Network nodes of interest are shown on a template brain in MNI space, with left lateral (left panel), right lateral (right panel), and axial views (center). ACC nodes appear in red, insula nodes in yellow, striatum nodes in blue, and amygdala nodes in green. The size of each node reflects its cumulative importance across 10 replications of predictive modeling. Nodes with cumulative importance >1 are labeled anatomically. The arrow next to each label indicates the sign of the regression coefficient for that node. Nodes that failed to appear in any of the 10 replications do not appear in this figure.

In the CC model, four predictors had cumulative importance >1 (**Figure 4**). The scatter plots (**Supplement 2**) illustrate that for the right rostral ACC, a higher CTQ was associated with a greater local influence on PBO than OXY, but for the left rostral ACC and right anterior-ventral insula, a higher CTQ was associated with a greater local influence on OXY than PBO. Greater head motion was associated with more years of cocaine use.

For all of the final models, VIFs were less than 2 for all predictors, indicating no collinearity issues, so all variables were retained.

## Discussion

The overall goal of this study was to discover how OXY changes functional network organization in men and women with CUD and to isolate network profiles that are associated with severity of cocaine use and childhood trauma. OXY induced increases in connectivity differently in men and women with CUD. In women, OXY increased local influence of the right accumbens and increased global influence of the right dorsal ACC. In men, OXY increased local influence of the left dorsal amygdala and right ACC.

The first hypothesis that childhood trauma would be associated with OXY-related connectivity changes in the amygdala was not strongly supported. Network profiles associated with individual variations in childhood trauma for females did not include amygdala nodes, and modeling of network profiles in males did not reliably yield significant models. Although the amygdala was not implicated in individual variations in childhood trauma, OXY increased local influence (CC) of the left dorsal amygdala in men. In addition, a higher global influence of this same amygdala region was associated with higher stress ratings in men on PBO. Although the functions of different amygdala nuclei in higher-level human behaviors is still debated, the dorsal (i.e., superficial) amygdala is involved in emotion processing, whereas the other amygdala nuclei play a role in fear, anxiety, and fear conditioning (27). Consequently, the association between dorsal amygdala global influence and stress ratings on PBO (in males) may reflect current emotional state rather than trauma history. In the PBO condition, a higher reported stress in males was associated with stronger global influence and more widespread connectivity of the left dorsal amygdala in males. In other words, the amygdala is exerting a stronger influence on other brain circuitry in the PBO condition, especially for males reporting more stress. Notably, OXY increased *local* influence of the left dorsal amygdala in males, suggesting that OXY shifts the influence of the left dorsal amygdala from global to more local and segregated from other brain circuitry. This shift on OXY may reflect an adaptive process, whereby stress-related amygdala activity is reduced.

The second hypothesis that years of cocaine use would be more strongly associated with OXY connectivity changes in the striatum was also not strongly supported given that none of the striatum nodes in males or females had cumulative importance that exceeded 1. However, OXY increased local influence of the right nucleus accumbens in females, indicating that it was influenced by OXY in females. Bethlehem and colleagues (28) similarly showed that OXY increased connectivity of the striatum with a broad network of brain regions in non-SUD women.

The third hypothesis that the major nodes of the salience network (insula, ACC) were expected to be associated with both childhood trauma and years of cocaine use was largely confirmed. ACC nodes predicted CTQ scores in females, and ACC and insula nodes predicted years of cocaine use in both males and females. The ACC was an important predictor in all models while the insula was an important predictor in one model (prediction of years of use in females). Local influence of the right ACC also increased on OXY in men, and global influence of the right dorsal ACC increased on OXY in women.

The fourth hypothesis was that network profiles associated with childhood trauma and years of cocaine use would be different between men and women. Stress circuitry (e.g., amygdala nodes) was expected to be more influential on network organization in females, whereas reward circuitry (e.g., striatum nodes) was expected to be more influential on network organization in males. Whereas the network profiles were indeed different between males and females, the amygdala was an important predictor of cocaine use in males rather than females (and was modulated by OXY in males), and the striatum was not an important predictor for either males or females, but the right accumbens was modulated by OXY in females.

The finding that amygdala connectivity was modulated by OXY, was associated with stress ratings under PBO, and was a significant component in the network profile for years of cocaine use in males but not females was not predicted. However, preclinical studies have reported that male rodents show greater OXY receptor binding in the amygdala than females, which is also modulated by breeding status in males (74). In addition, maltreated female adolescent rodents show significantly decreased OXY receptor binding in the amygdala compared to female controls (75). Although caution should be taken when translating preclinical findings to human study results, it is possible that the more prominent role for amygdala connectivity in CUD males in the present study is driven by higher OXY receptor binding in males and lower OXY receptor binding in females, particularly in those reporting more severe childhood trauma. This speculation, however, would need to be tested more directly in humans in future studies.

The predominant finding of the present study was that the salience network emerged as a critical component for OXY-induced changes in network profiles for childhood trauma and cocaine use in both males and females. Moreover, the ACC (rather than the insula) was the most prominent component in all models. The ACC is a critical node in the salience network that is functionally coupled to the insula. The ACC serves to influence external behaviors and motoric responses based on input from the insula (76), which processes interoceptive information and internal autonomic states (77). Given that the present study examined intrinsic connectivity (i.e., resting state) in the absence of external environmental input, the most salient information to be processed by subjects likely originated from internal bodily states. This may explain why the salience network was the primary influence on network organization. Had this study used external stimuli that could trigger reward responses, craving, or stress reactivity, the amygdala and striatum may have exerted a stronger influence on network organization.

Another potential explanation for the predominance of ACC nodes in influencing network organization is that the ACC is rich in OXY receptors (25). Because the present analysis focused on change in network connectivity related to OXY administration, those nodes that fall within brain regions with OXY receptors may have dominated network organization compared to regions that have fewer OXY receptors in humans, such as the striatum (25). It should be noted that the amygdala is also rich in OXY receptors, and this brain region emerged as an influential node in network profiles for individual variations in years of cocaine use in males. In addition, the exploratory analysis of subjective stress before scanning showed that higher reported stress was associated with greater global influence (EC) of the left dorsal amygdala in males in the PBO condition. These findings indicate that the amygdala may be an important locus for attenuating stress response in CUD males.

Wilcox and colleagues (17) have suggested that RSFC may be an important biomarker for treatment targets in SUDs. In their review of RSFC studies in SUD, they concluded that reduced connectivity between the salience network and executive control network and reduced connectivity within the executive control network are the most promising treatment targets for SUD. The present study has shown that OXY-related connectivity changes in components of the salience network, ACC, and insula are important for understanding individual variations in childhood trauma severity and cocaine use severity. Consequently, the present findings are consistent with the suggestion that the salience network is a potential treatment target.

It should be noted that associations between OXY-induced connectivity changes and childhood trauma or cocaine use severity were not universally in a single direction. In other words, higher cocaine use and greater childhood trauma were associated with both increases and decreases in connectivity because of OXY relative to PBO. Because this analysis considered a node’s relation to all other nodes in the network, it is reasonable that connectivity in one region could increase on OXY, whereas connectivity in another region could decrease. This is particularly true for graph-theory measures like CC and eigenvector centrality, which consider not only the direct connections to a node but also the connections of the connected nodes.

The two graph-theory properties examined here represent different aspects of network organization—local influence (CC) versus global influence (EC) of a node on the whole-brain network. CC has been investigated in prior rsfMRI studies of SUD (78–83), and only one study has examined EC in smokers (83). In the present study, both properties showed utility in characterizing network profiles for CTQ and years of cocaine use in CUD, but EC explained more variance across models and replications. The present findings demonstrate that EC is a potentially more useful graph-theory measure to consider when characterizing network profiles associated with individual differences in CUD. However, CC was more sensitive to changes in RSFC because of OXY.

### Limitations

One potential limitation of the present study is that we did not examine executive control network connectivity directly but focused instead on the influence of salience network, amygdala, and striatum nodes on intrinsic network organization. This could be viewed as a missed opportunity given a recent review suggesting that executive control network connectivity is a promising treatment target for SUD (17). However, the reason to limit the number of network nodes in the analysis was to avoid overfitting with automatic linear modeling. Nevertheless, the graph-theory measures used in this study reflect the connectivity of a given node with the entire brain, including frontal regions, thereby allowing for more specific hypotheses involving frontal cortex connectivity to be tested in future investigations.

The present analysis took several approaches to minimize contributions of head motion to graph-theory measures of connectivity (i.e., elimination of data sets with excessive head motion, temporal censoring, inclusion of six rigid-body head motion parameters as nuisance variables), and none of the graph-theory measures in individual nodes of interest was correlated with head motion. Therefore, the effects of head motion did not contaminate the measures of connectivity. Nevertheless, there are many other approaches to head-motion nuisance regression that are more stringent than the approach used in the present study [e.g., Ref. (55)], which could be considered a limitation. In addition, head motion emerged as a significant predictor of years of cocaine use in the final models that resulted from ALM. These findings indicate that head motion was associated with the outcome variable years of cocaine use. However, this association was different in males and females. For males, more years of cocaine use was associated with reduced head motion, but for females, more years of cocaine use was associated with increased head motion. The reason for this gender-specific divergence is not immediately apparent, but the present findings suggest that the extent of head motion is linked to individual variations in cocaine use and should probably be included in analyses even when head motion effects on connectivity are minimized.

Another potential limitation is that several substance use characteristics were not considered in the analyses but could be additional influences on changes in connectivity because of OXY. For example, positive THC tests and length of abstinence period before scanning could all affect resting-state connectivity and change in connectivity because of OXY. Future studies with larger samples should examine the influence of these substance use variables on OXY treatment response in CUD.

## Conclusion

In conclusion, this study adds to the evidence suggesting that RSFC may be an important biomarker in identifying treatment targets in SUDs. Salience network regions, especially the ACC, emerged as primary loci for OXY-induced changes in connectivity in both men and women with CUD, whereas the amygdala was an additional important locus for OXY response in males with CUD. These brain regions may serve as potential target areas for future OXY-based treatments. In addition, the present findings suggest that treatment strategies for CUD need to consider gender differences in OXY response.

## Ethics Statement

This study was carried out in accordance with the recommendations of Good Clinical Practice Guidelines and the Declaration of Helsinki with written informed consent from all subjects. All subjects gave written informed consent in accordance with the Declaration of Helsinki. The protocol was approved by the Medical University of South Carolina Institutional Review Board.

## Author Contributions

NB, KB, JJ, AM-C, and MM-S contributed to the design and conduct of the study. NB and JJ supervised and conducted the data analysis with contributions from KB, CC, AM-C, and BV. NB, KB, JJ, AM-C, and BS were involved in the interpretation of the data. JJ wrote the first draft of the manuscript. NB, KB, AM-C, BS, and BV helped write sections of and edited the manuscript.

## Funding

This study was sponsored by National Institute on Drug Abuse grants P50DA016511 and P50DA016511-S1 (KB) and K23DA045099 (BS), with additional support from the National Center for Advancing Translational Sciences grant UL1TR001450 (KB).

## Conflict of Interest Statement

The authors declare that the research was conducted in the absence of any commercial or financial relationships that could be construed as a potential conflict of interest.

## Footnotes

1
www.fmrib.ox.ac.uk/fsl

2
https://doi.org/10.5281/zenodo.1403924

3
http://strimmerlab.org/software/corpcor

## References

[1] Seedat S Scott KM Angermeyer MC Berglund P Bromet EJ Brugha TS Cross-national associations between gender and mental disorders in the World Health Organization World Mental Health Surveys. Arch Gen Psychiatry (2009) 66:785–95. 10.1001/archgenpsychiatry.2009.36 PMC2810067 19581570

[2] Becker JB Perry AN Westenbroek C Sex differences in the neural mechanisms mediating addiction: a new synthesis and hypothesis. Biol Sex Differ (2012) 3:14. 10.1186/2042-6410-3-14 22676718 PMC3724495

[3] Sutherland MT Mchugh MJ Pariyadath V Stein EA Resting state functional connectivity in addiction: lessons learned and a road ahead. Neuroimage (2012) 62:2281–95. 10.1016/j.neuroimage.2012.01.117 PMC3401637 22326834

[4] Lu H Stein EA Resting state functional connectivity: its physiological basis and application in neuropharmacology. Neuropharmacology (2014) 84:79–89. 10.1016/j.neuropharm.2013.08.023 24012656

[5] Hu Y Salmeron BJ Gu H Stein EA Yang Y Impaired functional connectivity within and between frontostriatal circuits and its association with compulsive drug use and trait impulsivity in cocaine addiction. JAMA Psychiatry (2015) 72:584–92. 10.1001/jamapsychiatry.2015.1 25853901

[6] Contreras-Rodriguez O Albein-Urios N Vilar-Lopez R Perales JC Martinez-Gonzalez JM Fernandez-Serrano MJ Increased corticolimbic connectivity in cocaine dependence versus pathological gambling is associated with drug severity and emotion-related impulsivity. Addict Biol (2016) 21:709–18. 10.1111/adb.12242 25818325

[7] Zhang S Wang W Zhornitsky S Li CR Resting state functional connectivity of the lateral and medial hypothalamus in cocaine dependence: an exploratory study. Front Psychiatry (2018) 9:344. 10.3389/fpsyt.2018.00344 30100886 PMC6072838

[8] Kelly C Zuo XN Gotimer K Cox CL Lynch L Brock D Reduced interhemispheric resting state functional connectivity in cocaine addiction. Biol Psychiatry (2011) 69:684–92. 10.1016/j.biopsych.2010.11.022 PMC3056937 21251646

[9] Mchugh MJ Demers CH Braud J Briggs R Adinoff B Stein EA Striatal-insula circuits in cocaine addiction: implications for impulsivity and relapse risk. Am J Drug Alcohol Abuse (2013) 39:424–32. 10.3109/00952990.2013.847446 24200212

[10] Berlingeri M Losasso D Girolo A Cozzolino E Masullo T Scotto M Resting state brain connectivity patterns before eventual relapse into cocaine abuse. Behav Brain Res (2017) 327:121–32. 10.1016/j.bbr.2017.01.002 28057531

[11] Mchugh MJ Demers CH Salmeron BJ Devous MD Sr. Stein EA Adinoff B Cortico-amygdala coupling as a marker of early relapse risk in cocaine-addicted individuals. Front Psychiatry (2014) 5:16. 10.3389/fpsyt.2014.00016 24578695 PMC3936467

[12] Adinoff B Gu H Merrick C Mchugh M Jeon-Slaughter H Lu H Basal hippocampal activity and its functional connectivity predicts cocaine relapse. Biol Psychiatry (2015) 78:496–504. 10.1016/j.biopsych.2014.12.027 25749098 PMC5671769

[13] Contreras-Rodriguez O Albein-Urios N Perales JC Martinez-Gonzalez JM Vilar-Lopez R Fernandez-Serrano MJ Cocaine-specific neuroplasticity in the ventral striatum network is linked to delay discounting and drug relapse. Addiction (2015) 110:1953–62. 10.1111/add.13076 26212416

[14] Geng X Hu Y Gu H Salmeron BJ Adinoff B Stein EA Salience and default mode network dysregulation in chronic cocaine users predict treatment outcome. Brain (2017) 140:1513–24. 10.1093/brain/awx036 PMC6075550 28334915

[15] Mccarthy JM Zuo CS Shepherd JM Dias N Lukas SE Janes AC Reduced interhemispheric executive control network coupling in men during early cocaine abstinence: a pilot study. Drug Alcohol Depend (2017) 181:1–4. 10.1016/j.drugalcdep.2017.09.009 29017089 PMC5683918

[16] Mchugh MJ Gu H Yang Y Adinoff B Stein EA Executive control network connectivity strength protects against relapse to cocaine use. Addict Biol (2017) 22:1790–801. 10.1111/adb.12448 27600492

[17] Wilcox CE Abbott CC Calhoun VD Alterations in resting-state functional connectivity in substance use disorders and treatment implications. Prog Neuropsychopharmacol Biol Psychiatry (2019) 91:79–93. 10.1016/j.pnpbp.2018.06.011 29953936 PMC6309756

[18] Gawrysiak MJ Jagannathan K Regier P Suh JJ Kampman K Vickery T Unseen scars: cocaine patients with prior trauma evidence heightened resting state functional connectivity (RSFC) between the amygdala and limbic-striatal regions. Drug Alcohol Depend (2017) 180:363–70. 10.1016/j.drugalcdep.2017.08.035 PMC5648604 28957777

[19] Wismer Fries AB Ziegler TE Kurian JR Jacoris S Pollak SD Early experience in humans is associated with changes in neuropeptides critical for regulating social behavior. Proc Natl Acad Sci U S A (2005) 102:17237–40. 10.1073/pnas.0504767102 PMC1287978 16303870

[20] Heim C Bradley B Mletzko TC Deveau TC Musselman DL Nemeroff CB Effect of childhood trauma on adult depression and neuroendocrine function: sex-specific moderation by CRH receptor 1 gene. Front Behav Neurosci (2009) 3:41. 10.3389/neuro.08.041.2009 20161813 PMC2821197

[21] Lee MR Rohn MC Tanda G Leggio L Targeting the oxytocin system to treat addictive disorders: rationale and progress to date. CNS Drugs (2016) 30:109–23. 10.1007/s40263-016-0313-z PMC4815424 26932552

[22] Kirsch P Esslinger C Chen Q Mier D Lis S Siddhanti S Oxytocin modulates neural circuitry for social cognition and fear in humans. J Neurosci (2005) 25:11489–93. 10.1523/JNEUROSCI.3984-05.2005 PMC6725903 16339042

[23] Labuschagne I Phan KL Wood A Angstadt M Chua P Heinrichs M Oxytocin attenuates amygdala reactivity to fear in generalized social anxiety disorder. Neuropsychopharmacology (2010) 35:2403–13. 10.1038/npp.2010.123 PMC3055328 20720535

[24] Koch SB Van Zuiden M Nawijn L Frijling JL Veltman DJ Olff M Intranasal oxytocin normalizes amygdala functional connectivity in posttraumatic stress disorder. Neuropsychopharmacology (2016) 41:2041–51. 10.1038/npp.2016.1 PMC4908648 26741286

[25] Boccia ML Petrusz P Suzuki K Marson L Pedersen CA Immunohistochemical localization of oxytocin receptors in human brain. Neuroscience (2013) 253:155–64. 10.1016/j.neuroscience.2013.08.048 24012742

[26] Dumais KM Bredewold R Mayer TE Veenema AH Sex differences in oxytocin receptor binding in forebrain regions: correlations with social interest in brain region- and sex- specific ways. Horm Behav (2013) 64:693–701. 10.1016/j.yhbeh.2013.08.012 24055336

[27] Seeley SH Chou YH O’connor MF Intranasal oxytocin and OXTR genotype effects on resting state functional connectivity: a systematic review. Neurosci Biobehav Rev (2018) 95:17–32. 10.1016/j.neubiorev.2018.09.011 30243577

[28] Bethlehem RAI Lombardo MV Lai MC Auyeung B Crockford SK Deakin J Intranasal oxytocin enhances intrinsic corticostriatal functional connectivity in women. Transl Psychiatry (2017) 7:e1099. 10.1038/tp.2017.72 28418398 PMC5416709

[29] Brodmann K Gruber O Goya-Maldonado R Intranasal oxytocin selectively modulates large-scale brain networks in humans. Brain Connect (2017) 7:454–63. 10.1089/brain.2017.0528 PMC5647506 28762756

[30] Zhao Z Ma X Geng Y Zhao W Zhou F Wang J Oxytocin differentially modulates specific dorsal and ventral striatal functional connections with frontal and cerebellar regions. Neuroimage (2019) 184:781–9. 10.1016/j.neuroimage.2018.09.067 30266264

[31] Connors GJ Maisto SA Zywiak WH Male and female alcoholics’ attributions regarding the onset and termination of relapses and the maintenance of abstinence. J Subst Abuse (1998) 10:27–42. 10.1016/S0899-3289(99)80138-2 9720004

[32] Terry-Mcelrath YM O’malley PM Johnston LD Reasons for drug use among American Youth by consumption level, gender, and race/ethnicity: 1976-2005. J Drug Issues (2009) 39:677–714. 10.1177/002204260903900310 20628558 PMC2902005

[33] Kilts CD Schweitzer JB Quinn CK Gross RE Faber TL Muhammad F Neural activity related to drug craving in cocaine addiction. Arch Gen Psychiatry (2001) 58:334–41. 10.1001/archpsyc.58.4.334 11296093

[34] Kilts CD Gross RE Ely TD Drexler KP The neural correlates of cue-induced craving in cocaine-dependent women. Am J Psychiatry (2004) 161:233–41. 10.1176/appi.ajp.161.2.233 14754771

[35] Potenza MN Hong KI Lacadie CM Fulbright RK Tuit KL Sinha R Neural correlates of stress-induced and cue-induced drug craving: influences of sex and cocaine dependence. Am J Psychiatry (2012) 169:406–14. 10.1176/appi.ajp.2011.11020289 PMC3690485 22294257

[36] Teicher MH Andersen SL Polcari A Anderson CM Navalta CP Developmental neurobiology of childhood stress and trauma. Psychiatr Clin North Am (2002) 25:397–426. , vii-viii. 10.1016/S0193-953X(01)00003-X 12136507

[37] Stevens JS Van Rooij SJH Jovanovic T Developmental contributors to trauma response: the importance of sensitive periods, early environment, and sex differences. Curr Top Behav Neurosci (2018) 38:1–22. 10.1007/7854_2016_38 27830573 PMC5425320

[38] Frijling JL Van Zuiden M Koch SB Nawijn L Veltman DJ Olff M Intranasal oxytocin affects amygdala functional connectivity after trauma script-driven imagery in distressed recently trauma-exposed individuals. Neuropsychopharmacology (2016) 41:1286–96. 10.1038/npp.2015.278 PMC4793112 26346640

[39] Koob GF Volkow ND Neurocircuitry of addiction. Neuropsychopharmacology (2010) 35:217–38. 10.1038/npp.2009.110 PMC2805560 19710631

[40] Peters SK Dunlop K Downar J Cortico-striatal-thalamic loop circuits of the salience network: a central pathway in psychiatric disease and treatment. Front Syst Neurosci (2016) 10:104. 10.3389/fnsys.2016.00104 28082874 PMC5187454

[41] Sheehan DV Lecrubier Y Sheehan KH Amorim P Janavs J Weiller E The Mini-International Neuropsychiatric Interview (M.I.N.I).: the development and validation of a structured diagnostic psychiatric interview for DSM-IV and ICD-10. J Clin Psychiatry (1998) 59 Suppl 20:22–33. ;quiz 34-57. 9881538

[42] First MB Spitzer RL Gibbon M Williams JB (2002). “Structured clinical interview for DSM-IV-TR axis I disorders, research version, patient edition”. SCID-I/P).

[43] Sobell LC Sobell MB Timeline follow-back: A technique for assessing self-reported alcohol consumption. In: Litten RZ Allen JP, editors. Measuring alcohol consumption: Psychosocial and biomedical methods. Humana Press (1992). p. 41–72. 10.1007/978-1-4612-0357-5_3

[44] Bernstein DP Fink L Childhood Trauma Questionnaire: A retrospective self-report manual. San Antonio, TX: The Psychological Corporation (1998).

[45] Childress AR Mclellan AT O’brien CP Conditioned responses in a methadone population: a comparison of laboratory, clinic, and natural settings. J Subst Abuse Treat (1986) 3:173–9. 10.1016/0740-5472(86)90018-8 3806730

[46] Spielberger CD Gorsuch RL Lushene R Vagg PR Jacobs GA Manual for the State-Trait Anxiety Inventory. Palo Alto, CA: Consulting Psychologists Press (1983).

[47] Heinrichs M Baumgartner T Kirschbaum C Ehlert U Social support and oxytocin interact to suppress cortisol and subjective responses to psychosocial stress. Biol Psychiatry (2003) 54:1389–98. 10.1016/S0006-3223(03)00465-7 14675803

[48] Ditzen B Schaer M Gabriel B Bodenmann G Ehlert U Heinrichs M Intranasal oxytocin increases positive communication and reduces cortisol levels during couple conflict. Biol Psychiatry (2009) 65:728–31. 10.1016/j.biopsych.2008.10.011 19027101

[49] Kubzansky LD Mendes WB Appleton A Block J Adler GK Protocol for an experimental investigation of the roles of oxytocin and social support in neuroendocrine, cardiovascular, and subjective responses to stress across age and gender. BMC Public Health (2009) 9:481. 10.1186/1471-2458-9-481 20025778 PMC2805640

[50] Mcrae-Clark AL Baker NL, Moran-Santa Maria M Brady KT Effect of oxytocin on craving and stress response in marijuana-dependent individuals: a pilot study. Psychopharmacology (2013) 228:623–31. 10.1007/s00213-013-3062-4 PMC3729589 23564179

[51] Flanagan JC Baker NL Mcrae-Clark AL Brady KT Moran-Santa Maria MM Effects of adverse childhood experiences on the association between intranasal oxytocin and social stress reactivity among individuals with cocaine dependence. Psychiatry Res (2015) 229:94–100. 10.1016/j.psychres.2015.07.064 26231584 PMC4546857

[52] Heinrichs M Von Dawans B Domes G Oxytocin, vasopressin, and human social behavior. Front Neuroendocrinol (2009) 30:548–57. 10.1016/j.yfrne.2009.05.005 19505497

[53] Holm S A simple sequentially rejective multiple test procedure. Scand J Stat (1979), 65–70.

[54] Benjamini Y Hochberg Y Controlling the false discovery rate: a practical and powerful approach to multiple testing. J R Stat Soc Series B Stat Methodol (1995) 57:289–300. 10.1111/j.2517-6161.1995.tb02031.x

[55] Satterthwaite TD Elliott MA Gerraty RT Ruparel K Loughead J Calkins ME An improved framework for confound regression and filtering for control of motion artifact in the preprocessing of resting-state functional connectivity data. Neuroimage (2013) 64:240–56. 10.1016/j.neuroimage.2012.08.052 PMC3811142 22926292

[56] Cox RW AFNI: software for analysis and visualization of functional magnetic resonance neuroimages. Comput Biomed Res (1996) 29:162–73. 10.1006/cbmr.1996.0014 8812068

[57] Power JD Barnes KA Snyder AZ Schlaggar BL Petersen SE Spurious but systematic correlations in functional connectivity MRI networks arise from subject motion. Neuroimage (2012) 59:2142–54. 10.1016/j.neuroimage.2011.10.018 PMC3254728 22019881

[58] Rcoreteam (2018). “R: A language and environment for statistical computing”. 3.5.1 ed (Vienna, Austria: R Foundation for Statistical Computing).

[59] Ledoit O Wolf M Improved estimation of the covariance matrix of stock retruns with an application to portfolio science. J Empir Financ (2003) 10:603–21. 10.1016/S0927-5398(03)00007-0

[60] Schafer J Strimmer K A shrinkage approach to large-scale covariance matrix estimation and implications for functional genomics. Stat. Appl. Genet. Mol. Biol. (2005) 4. 10.2202/1544-6115.1175 16646851

[61] Opgen-Rhein R Strimmer K Accurate ranking of differentially expressed genes by a distribution-free shrinkage approach. Stat Appl Genet Mol Biol (2007) 6Article9. 10.2202/1544-6115.1252 17402924

[62] Lohmann G Margulies DS Horstmann A Pleger B Lepsien J Goldhahn D Eigenvector centrality mapping for analyzing connectivity patterns in fMRI data of the human brain. PLoS ONE (2010) 5:e10232. 10.1371/journal.pone.0010232 20436911 PMC2860504

[63] Fornito A Harrison BJ Zalesky A Simons JS Competitive and cooperative dynamics of large-scale brain functional networks supporting recollection. Proc Natl Acad Sci U S A (2012) 109:12788–93. 10.1073/pnas.1204185109 PMC3412011 22807481

[64] Watts DJ Strogatz SH Collective dynamics of ‘small-world’ networks. Nature (1998) 393:440–2. 10.1038/30918 9623998

[65] Rubinov M Sporns O Complex network meansures of brain connectivity: uses and interpretations. Comput Models Brain (2010) 52:1059–69. 10.1016/j.neuroimage.2009.10.003 19819337

[66] Costantini G Perugini M Generalization of clustering coefficients to signed correlation networks. PLoS One (2014) 9:e88669. 10.1371/journal.pone.0088669 24586367 PMC3931641

[67] Xia M Wang J He Y BrainNet Viewer: a network visualization tool for human brain connectomics. PLoS ONE (2013) 8:e68910. 10.1371/journal.pone.0068910 23861951 PMC3701683

[68] Arnsten AF Stress signalling pathways that impair prefrontal cortex structure and function. Nat Rev Neurosci (2009) 10:410–22. 10.1038/nrn2648 PMC2907136 19455173

[69] Lupien SJ Mcewen BS Gunnar MR Heim C Effects of stress throughout the lifespan on the brain, behaviour and cognition. Nat Rev Neurosci (2009) 10:434–45. 10.1038/nrn2639 19401723

[70] Hart H Rubia K Neuroimaging of child abuse: a critical review. Front Hum Neurosci (2012) 6:52. 10.3389/fnhum.2012.00052 22457645 PMC3307045

[71] Van Harmelen AL Hauber K Gunther Moor B Spinhoven P Boon AE Crone EA Childhood emotional maltreatment severity is associated with dorsal medial prefrontal cortex responsivity to social exclusion in young adults. PLoS One (2014) 9:e85107. 10.1371/journal.pone.0085107 24416347 PMC3885678

[72] Schatzoff M Tsao R Fienberg S Efficient computing of all possible regressions. Technometrics (1968) 10:769–79. 10.2307/1267458

[73] Hair J Black WC Babin BJ Anderson RE Multivariate Data Analysis. Upper Saddle River, NJ: Pearson Education International (2010).

[74] Mooney SJ Coen CW Holmes MM Beery AK Region-specific associations between sex, social status, and oxytocin receptor density in the brains of eusocial rodents. Neuroscience (2015) 303:261–9. 10.1016/j.neuroscience.2015.06.043 26143015

[75] Hill KT Warren M Roth TL The influence of infant-caregiver experiences on amygdala Bdnf, OXTr, and NPY expression in developing and adult male and female rats. Behav Brain Res (2014) 272:175–80. 10.1016/j.bbr.2014.07.001 PMC4103656 25011012

[76] Menon V Uddin LQ Saliency, switching, attention and control: a network model of insula function. Brain Struct Funct (2010) 214:655–67. 10.1007/s00429-010-0262-0 PMC2899886 20512370

[77] Craig AD Forebrain emotional asymmetry: a neuroanatomical basis? Trends Cogn Sci (2005) 9:566–71. 10.1016/j.tics.2005.10.005 16275155

[78] Breckel TP Thiel CM Giessing C The efficiency of functional brain networks does not differ between smokers and non-smokers. Psychiatry Res (2013) 214:349–56. 10.1016/j.pscychresns.2013.07.005 24144504

[79] Giessing C Thiel CM Alexander-Bloch AF Patel AX Bullmore ET Human brain functional network changes associated with enhanced and impaired attentional task performance. J Neurosci (2013) 33:5903–14. 10.1523/JNEUROSCI.4854-12.2013 PMC6618923 23554472

[80] Jiang G Wen X Qiu Y Zhang R Wang J Li M Disrupted topological organization in whole-brain functional networks of heroin-dependent individuals: a resting-state FMRI study. PLoS ONE (2013) 8:e82715. 10.1371/journal.pone.0082715 24358220 PMC3866189

[81] Lin F Wu G Zhu L Lei H Altered brain functional networks in heavy smokers. Addict Biol (2015) 20:809–19. 10.1111/adb.12155 24962385

[82] Wang Z Suh J Li Z Li Y Franklin T O’brien C A hyper-connected but less efficient small-world network in the substance-dependent brain. Drug Alcohol Depend (2015) 152:102–8. 10.1016/j.drugalcdep.2015.04.015 PMC4458212 25957794

[83] Moran-Santa Maria MM Vanderweyen DC Camp CC Zhu X Mckee SA Cosgrove KP Network analysis of intrinsic functional brain connectivity in male and female adult smokers: a preliminary study. Nicotine Tob Res (2018) 20:810–8. 10.1093/ntr/ntx206. PMC5991199 29059410

