# Supplementary material for: Oxytocin-Induced Changes in Intrinsic Network Connectivity in Cocaine Use Disorder: Modulation by Gender, Childhood Trauma, and Years of Use
Source: Front Psychiatry. 2019 Jul 19;10:502. doi: 10.3389/fpsyt.2019.00502 (PMC6658612; doi:10.3389/fpsyt.2019.00502)
Supplement: Supplementary file 1 [file DataSheet_1.docx]

***Supplement 1***

***Results from the generalized linear model analyses***

Table S1-1: Effect of node, treatment (tx), gender, head motion, CTQ and YRSUSE on Clustering Coefficient

Effect Wald Chi-Square df Sig.

node 279.102 19 .000

tx 0.742 1 .389

Gender 6.119 1 .013

CTQ 4.171 1 .041

YRSUSE 0.969 1 .325

visit 0.003 1 .956

head motion 24.542 1 .000

node * tx 48.744 19 .000

tx * Gender 0.138 1 .710

tx * CTQ 0.085 1 .771

tx * YRSUSE 2.795 1 .095

node * tx * Gender 104.304 38 .000

node * tx * CTQ 134.163 38 .000

node * tx * YRSUSE 92.882 38 .000

node * tx * Gender * CTQ 241.577 40 .000

node * tx * Gender * YRSUSE 93.091 40 .000

Table S1-2: Effect of node, treatment (tx), gender, head motion, CTQ and YRSUSE on

on Eigenvector Centrality

Effect Wald Chi-Square df Sig.

node 49.212 19 .000

tx 0.156 1 .693

Gender 0.003 1 .959

CTQ 1.042 1 .307

YRSUSE 0.414 1 .520

Visit 1.085 1 .298

head motion 1.477 1 .224

node * tx 13.717 19 .800

tx * Gender 7.732 1 .005

tx * CTQ 0.195 1 .659

tx * YRSUSE 2.177 1 .140

node * tx * Gender 236.694 38 .000

node * tx * CTQ 83.660 38 .000

node * tx * YRSUSE 201.575 38 .000

node * tx * Gender * CTQ 195.726 40 .000

node * tx * Gender * YRSUSE 187.051 40 .000
