# Supplementary material for: Oxytocin-Induced Changes in Intrinsic Network Connectivity in Cocaine Use Disorder: Modulation by Gender, Childhood Trauma, and Years of Use
Source: Front Psychiatry. 2019 Jul 19;10:502. doi: 10.3389/fpsyt.2019.00502 (PMC6658612; doi:10.3389/fpsyt.2019.00502)
Supplement: Supplementary file 2 [file DataSheet_2.docx]

***Supplement 2***

***Information on predictor variables for each final model***

Table S2-1: Network profile for CTQ in Males based on clustering coefficient

Cumulative Average Regression Average

Predictor Importance Coefficient p-value

Right dorsal ACC 0.090 1727.56 0.68

Right posterior Insula 0.604 -5722.48 0.31

Right mid Insula 1.757 -11233.60 0.05

Left ACC 0.010 2895.72 0.70

Left rostral ACC 0.116 1946.93 0.66

Right rostral ACC 2.308 9960.03 0.04

Left anterior Insula 0.003 -1062.37 0.83

Right anterior Insula 0.023 595.33 0.75

Right anterior ventral Insula n/a n/a n/a

Right ACC 2.133 -8217.68 0.13

Left dorsal Amygdala 0.199 -4880.46 0.31

Right dorsal Amygdala 0.071 2311.13 0.65

Left medial Amygdala 1.309 6635.25 0.13

Right medial Amygdala 0.325 3382.97 0.45

Left ventrolateral Amygdala 0.058 -2550.32 0.57

Right ventrolateral Amygdala 0.136 -3240.82 0.44

Left Caudate 0.243 -3762.69 0.43

Right Caudate 0.014 -1323.16 0.78

Left Nucleus Accumbens 0.561 -7144.70 0.19

Right Nucleus Accumbens 0.014 2045.31 0.66

Head Motion 0.025 12.98 0.75

n/a: predictor did not appear in any of the 10 replications

Figure S2-1: Scatter Plots for Predictors with Cumulative Importance > 1 for network profile for childhood trauma in males. For all graphs, the x-axis represents the change in clustering coefficient due to oxytocin (placebo minus oxytocin difference score (PBO – OXY)). For all graphs, the y-axis represents the total CTQ score.

Table S2-2: Network profile for CTQ in Females based on eigenvector centrality

Cumulative Average Regression Average

Predictor Importance Coefficient p-value

Right dorsal ACC 2.874 -298.57 0.02

Right posterior Insula 0.256 169.83 0.22

Right mid Insula 0.597 151.09 0.21

Left ACC 0.163 -48.04 0.71

Left rostral ACC 1.288 -302.12 0.07

Right rostral ACC 0.515 -164.77 0.22

Left anterior Insula 0.000 -4.64 0.97

Right anterior Insula 0.717 178.79 0.11

Right anterior ventral Insula 0.498 201.39 0.12

Right ACC 1.306 203.43 0.06

Left dorsal Amygdala 0.154 125.39 0.30

Right dorsal Amygdala 0.676 131.80 0.19

Left medial Amygdala 0.072 28.60 0.29

Right medial Amygdala 0.028 0.62 0.52

Left ventrolateral Amygdala 0.390 149.98 0.18

Right ventrolateral Amygdala 0.090 -54.91 0.39

Left Caudate 0.024 -64.79 0.61

Right Caudate 0.219 -109.82 0.22

Left Nucleus Accumbens 0.032 -30.82 0.57

Right Nucleus Accumbens 0.102 -223.89 0.06

Head motion n/a n/a n/a

n/a: predictor did not appear in any of the 10 replications

Figure S2-2: Scatter Plots for Predictors with Cumulative Importance > 1 for network profile for childhood trauma in females. For all graphs, the x-axis represents the change in eigenvector centrality due to oxytocin (placebo minus oxytocin difference score (PBO – OXY)). For all graphs, the y-axis represents the total CTQ score.

Table S2-3: Network profile for YRSUSE in Males based on eigenvector centrality

Cumulative Average Regression Average

Predictor Importance Coefficient p-value

Right dorsal ACC 1.256 57.69 0.07

Right posterior Insula 0.019 -15.54 0.55

Right mid Insula n/a n/a n/a

Left ACC 0.027 9.00 0.71

Left rostral ACC 0.134 22.71 0.41

Right rostral ACC 0.028 -17.04 0.47

Left anterior Insula 0.075 37.52 0.43

Right anterior Insula 0.230 -44.16 0.28

Right anterior ventral Insula 0.137 -11.82 0.60

Right ACC 0.059 21.60 0.55

Left dorsal Amygdala 0.168 28.56 0.35

Right dorsal Amygdala 0.034 -19.65 0.53

Left medial Amygdala 4.682 -120.60 0.001

Right medial Amygdala 0.055 -1.13 0.62

Left ventrolateral Amygdala 0.196 -68.02 0.14

Right ventrolateral Amygdala 0.141 -8.15 0.46

Left Caudate 0.720 80.62 0.14

Right Caudate 0.189 -61.79 0.34

Left Nucleus Accumbens 0.211 46.57 0.17

Right Nucleus Accumbens 0.499 38.15 0.27

Head motion 1.124 -45.39 0.05

n/a: predictor did not appear in any of the 10 replications

Figure S2-3: Scatter Plots for Predictors with Cumulative Importance > 1 for network profile for YRSUSE in males. For the left 2 graphs, the x-axis represents the change in eigenvector centrality due to oxytocin (placebo minus oxytocin difference score (PBO – OXY)). For the right graph the x-axis represents the average head motion over both visits based on % of scrubbed time points. For all graphs, the y-axis represents the total years of cocaine use.

Table S2-4: Network profile for YRSUSE in Females based on clustering coefficient

Cumulative Average Regression Average

Predictor Importance Coefficient p-value

Right dorsal ACC 0.214 1959.01 0.50

Right posterior Insula 0.097 -2315.04 0.51

Right mid Insula 0.480 -4294.78 0.22

Left ACC n/a n/a n/a

Left rostral ACC 3.029 -7459.19 0.003

Right rostral ACC 1.740 4967.42 0.03

Left anterior Insula 0.053 754.62 0.72

Right anterior Insula 0.021 -1710.21 0.57

Right anterior ventral Insula 1.094 -6727.90 0.06

Right ACC 0.027 -1702.86 0.45

Left dorsal Amygdala 0.242 3734.79 0.28

Right dorsal Amygdala 0.048 965.22 0.58

Left medial Amygdala 0.207 -68.98 0.49

Right medial Amygdala 0.079 530.59 0.56

Left ventrolateral Amygdala 0.057 -2434.28 0.26

Right ventrolateral Amygdala 0.174 -3960.14 0.26

Left Caudate 0.110 926.55 0.54

Right Caudate 0.016 -469.00 0.77

Left Nucleus Accumbens 0.418 -2869.66 0.25

Right Nucleus Accumbens 0.013 1277.11 0.61

Head motion 1.879 52.09 0.03

n/a: predictor did not appear in any of the 10 replications

Figure S2-4: Scatter Plots for Predictors with Cumulative Importance > 1 for network profile for YRSUSE in females. For the left 2 graphs and upper right graph, the x-axis represents the change in clustering coefficient due to oxytocin (placebo minus oxytocin difference score (PBO – OXY)). For the lower right graph the x-axis represents the average head motion over both visits based on % of scrubbed time points. For all graphs, the y-axis represents the total years of cocaine use.
